# Frequency of Balanced-Meal Consumption and Frailty in Community-Dwelling Older Japanese: A Cross-Sectional Study

**DOI:** 10.2188/jea.JE20180076

**Published:** 2019-10-05

**Authors:** Yuri Yokoyama, Akihiko Kitamura, Mariko Nishi, Satoshi Seino, Yu Taniguchi, Hidenori Amano, Tomoko Ikeuchi, Shoji Shinkai

**Affiliations:** 1 Research Team for Social Participation and Community Health, Tokyo Metropolitan Institute of Gerontology, Tokyo, Japan; 2 Tokyo Metropolitan Institute of Gerontology, Tokyo, Japan

**Keywords:** frailty, balanced meals, Japanese diet, diet quality

## Abstract

**Background:**

Although meals that combine a staple food, main dish, and side dish (balanced meals) are recommended in Japan, the health effects of such meals are unclear. We investigated the association of frequency of eating balanced meals with frailty among community-dwelling older Japanese.

**Methods:**

We analyzed data from 912 persons aged 65 years or older who participated in the Hatoyama Cohort Study or Kusatsu Longitudinal Study. The frequency of eating two or more balanced meals daily was self-reported as ≤1 day/week, 2 or 3 days/week, 4 or 5 days/week, and daily. Frailty was defined as the presence of at least three, and pre-frailty as the presence of one or two, of the following criteria: weight loss, muscle weakness, exhaustion, slowness, and low physical activity. Adjusted logistic regression was used to study associations of frequency of balanced-meal consumption with frailty (prefrailty and frailty combined) and frailty criteria.

**Results:**

Participants reporting a frequency of balanced-meal consumption of ≤2 or 3 days/week had a higher prevalence of frailty (odds ratio [OR], 1.79; 95% confidence interval [CI], 1.21–2.64) than did those reporting a frequency of daily. Lower frequency of balanced-meal consumption was also associated with higher prevalences of weight loss (OR, 4.10; 95% CI, 1.90–8.85), exhaustion (OR, 6.35; 95% CI, 2.49–16.17), and low physical activity (OR, 1.92; 95% CI, 1.22–3.01).

**Conclusions:**

Our findings suggest that more frequent twice daily consumption of meals with a staple food, main dish, and side dish decreases the risks of prefrailty and frailty.

## INTRODUCTION

Frailty is a geriatric syndrome with multiple causes and contributors. It is characterized by diminished strength, endurance, and physical function—conditions that increase the risks of dependence and death.^1^ As populations age and longevity increases, the number of frail older people will rise, thus increasing burdens on the community and health care system. Therefore, effective strategies to prevent or delay frailty are urgently required.

Nutrition is a modifiable factor that may be important in the development of frailty and its components. Several studies reported associations of frailty and its components with specific nutrients and foods, such as protein,^2^^–^^5^ vitamin D,^2^^,^^6^^,^^7^ antioxidants nutrients,^2^^,^^6^^,^^8^ n-3 polyunsaturated fatty acids (PUFAs),^9^^–^^11^ fruit and vegetables,^12^ and dairy products (low-fat milk and yogurt).^13^ Furthermore, studies of the whole diet, in addition to analyses of specific nutrients and foods, have yielded a growing body of evidence indicating that the Mediterranean dietary pattern protects against frailty and its components.^14^^–^^16^ However, the Mediterranean diet differs greatly from traditional Japanese diets, and dietary patterns other than the Mediterranean diet may be equally or more effective for the Japanese population. Thus, it is important to identify Japanese dietary patterns that help reduce frailty risk.

Kobayashi et al reported that high protein, high dietary total antioxidant capacity, and their combination was inversely associated with the prevalence of frailty among older Japanese women.^4^^,^^17^^,^^18^ However, no study has examined the association between a characteristic Japanese dietary pattern and frailty. Meals combining a staple food, main dish, and side dish (hereafter referred to as “balanced meals”) are a major characteristic of the Japanese dietary pattern and are recommended in a Japanese dietary guideline.^19^ The Food and Nutrition chapter of “National Health Promotion Movement in the 21st Century” (Health Japan 21, Second Edition) suggests that “eating balanced meals with a staple food, main dish, and side dish at least twice a day” is an important indicator of longer healthy life expectancy and improved quality of life.^20^

Previous findings suggest that such meals are more balanced in nutrient intake and intakes of the various food groups.^21^^,^^22^ However, the health benefits of such a diet, including prevention or delay of frailty, have not been examined. This cross-sectional study examined the association of the frequency of balanced-meal consumption with frailty in community-dwelling older Japanese.

## METHODS

This cross-sectional analysis used data from the Hatoyama Cohort Study and Kusatsu Longitudinal Study.^23^^,^^24^ The study designs and protocols have been reported elsewhere.^23^^,^^24^ Briefly, the Hatoyama Cohort Study was a prospective cohort study, begun in 2010, of 742 community-dwelling adults aged 65 years or older living in the town of Hatoyama in Saitama Prefecture, Japan. The study sample was constructed using stratified random sampling, with participants classified by age and residential area. The Kusatsu Longitudinal Study was a longitudinal study, begun in 2002, of older adults living in the town of Kusatsu, a rural community in northwest Gunma Prefecture, Japan. The target population comprised National Health Insurance enrollees aged 65–74 years and adults enrolled in the Medical Insurance System for the Elderly Aged 75 Years or Older. Because common variables were collected in the 2016 survey of the Hatoyama Study and the 2017 survey of the Kusatsu Study, data for both time points were combined for the present study.

In total, 1,064 adults (357 from the 2016 survey of the Hatoyama study and 707 from the 2017 survey of the Kusatsu study) participated in the survey. Among 1,064 study participants, we excluded 109 who lacked complete data on frailty, 2 with no information on frequency of balanced-meal consumption, and 62 with missing data on covariates. Therefore, the final analytical sample comprised 912 participants. The excluded individuals were younger, more likely to be women, and less educated. However, frailty and frequency of balanced-meal consumption did not significantly differ between participants included in the study sample and those who were excluded.

The protocol of this study was approved by the Ethics Committee at Tokyo Metropolitan Institute of Gerontology, and written informed consent was obtained from all participants.

### Frailty

Frailty was defined in accordance with the Cardiovascular Health Study frailty index,^25^ modified to include criteria of weight loss, exhaustion, muscle weakness, slowness, and low physical activity. Weight loss was defined as a self-reported unintentional weight loss of ≥3 kg during the preceding 6 months.^26^ Exhaustion was defined as a response of, “I felt that everything I did was an effort” or “I could not get going”, on the Center for Epidemiologic Studies-Depression^27^ scale for “at least 3 to 4 days a week”. Muscle weakness was defined using maximum grip strength, measured in kilograms with a Smedley-type handheld dynamometer. In addition, weakness was defined using sex-specific cutoffs (men: <26 kg, women: <18 kg).^28^ Slowness was defined using usual gait speed, which was measured over a straight 11-m walkway marked with tape at 3 and 8 m. The time required to walk 5 m was measured, and gait speed (m/s) was calculated. Slowness was established according to a cutoff (<1.0 m/s).^28^ A previous Japanese study reported that these cut-off values for slowness (walking speed <1.0 m/s) and weakness (handgrip strength <26 kg for men and <18 kg for women) are appropriate criteria for assessment of physical frailty in Japanese elders.^28^ Low physical activity was identified by a response of “No” to the question, “Do you exercise regularly?” on the questionnaire. Frailty was defined as the presence of at least three, and pre-frailty as the presence of one or two, of the five criteria. Because of the low prevalence of frailty in the present study, we combined prefrail and frail states.

### Frequency of balanced-meal consumption

Participants were asked the following questions, which included brief descriptions of staple foods (*shushoku*), main dishes (*shusai*), and side dishes (*fukusai*): “How many days per week do you eat at least two meals a day with a staple food (*shushoku*), main dish (*shusai*), and side dish (*fukusai*)?” The frequency was self-reported as ≤1 day/week, 2 or 3 days/week, 4 or 5 days/week, and daily. The brief description was as follows: “*Shushoku* includes cereals, such as rice, bread, and noodles. *Shusai* includes dishes with fish, meat, eggs, or soy products and is the main dish. *Fukusai* includes dishes with vegetables, mushrooms, potatoes, and algae.” This question was used by the Ministry of Agriculture, Forestry and Fisheries in Japan in research on the present state of and consciousness of food education (*shokuiku*)^29^ and by the Ministry of Health, Labour and Welfare in Japan in the 2015 National Health and Nutrition Survey.^30^

### Other variables

In addition to the above variables, data were collected on covariates potentially associated with diet and frailty, including sex, age, study site, education level, living alone, smoking habit (never, former, or current smoker), drinking habit (never/rarely, sometimes, or every day), self-perceived chewing ability (can chew anything/almost anything or do not chew much), medical history (hypertension, hyperlipidemia, diabetes mellitus, heart disease, stroke, cancer, arthritis, and chronic obstructive pulmonary disease), and body mass index (computed as weight in kilograms divided by height in meters squared).

### Statistical analysis

The frequency of balanced-meal consumption was classified as daily, 4 or 5 days/week, and ≤2 or 3 days/week. To maximize statistical power, two frequency categories (≤1 day/week and 2 or 3 days/week) were combined into a single category (≤2 or 3 days/week) because few participants reported a frequency of ≤1 day/week.

Participant characteristics were compared in relation to balanced-meal consumption with weighted one-way analyses of variance, for continuous variables, and the Mantel–Haenszel chi-square test, for categorical variables. Multiple logistic regression was used to evaluate associations of balanced-meal consumption with frailty (prefrailty and frailty combined) and frailty criteria in the complete data (*n* = 912). The response category daily was used as the reference group in the analysis. Model 1 was adjusted for age, sex, and study site. Model 2 was additionally adjusted for education level (years), living alone, self-perceived chewing ability, smoking habit, drinking habit, body mass index, and medical history. The *P* value for the linear trend was estimated by modeling the frequency categories for balanced-meal consumption as a continuous variable. Analyses of the association of balanced-meal consumption with frailty (prefrailty and frailty combined) were performed separately in men and women. However, the associations of balanced-meal consumption with the components of frailty were analyzed in both sexes combined, because the prevalences of individual frailty components were too low for sex-stratified statistical analysis. We additionally analyzed data stratified by area (Hatoyama or Kusatsu).

Sensitivity analyses were conducted by using data from 1,064 participants. Multiple imputation was used to ensure that missing data did not introduce bias to results regarding the association between frequency of balanced-meal consumption and frailty. Multiple imputation was performed using five imputed datasets generated with a multiple imputation approach, under the assumption that data were missing at random.^31^ All analyses were performed with IBM SPSS Statistics version 23 (IBM Corp, Armonk, NY, USA).

## RESULTS

Of the 912 participants, 50.3% were women, and mean participant age was 75.6 (standard deviation, 5.8) years. The prevalences of the components of the frailty phenotype—weight loss, exhaustion, weakness, slowness, and low activity—were 4.1%, 3.8%, 18.2%, 8.7%, and 17.2%, respectively. The prevalences of frailty and prefrailty were 3.1% and 34.5%, respectively. In stratified analyses by sex, the prevalences of weight loss, exhaustion, weakness, slowness, and low activity were 5.7%, 4.6%, 13.2%, 7.7%, and 17.7% in men and 2.4%, 3.1%, 23.1%, 9.6%, and 16.8% in women, respectively. The prevalences of frailty and prefrailty were 2.4% and 34.2% in men and 3.7% and 34.9% in women, respectively.

Participant characteristics in relation to frequency of balanced-meal consumption are shown in Table 1. Both men and women who ate twice-daily balanced meals less frequently than 2 or 3 days/week were more likely to be living alone and to be unable to chew most foods. Men who ate twice-daily balanced meals less frequently than 2 or 3 days/week were less likely to drink and have hyperlipidemia and more likely to have arthritis and COPD. Women who ate twice-daily balanced meals less frequently than 2 or 3 days/week had less education, were more likely to smoke and have hypertension and heart disease, and were less likely to have arthritis.

**Table 1.** Participant characteristics in relation to frequency of twice-daily balanced-meal consumption

	Total	Men				Women			
	
		Frequency of twice-daily balanced-meal consumption^a^			*P* for trend^b^	Frequency of twice-daily balanced-meal consumption^a^			*P* for trend^b^
	
		Daily	4 or 5 d/wk	≤2 or 3 d/wk		Daily	4 or 5 d/wk	≤2 or 3 d/wk	
Number of participants	912	286	72	95		309	79	71	
Age, years, mean (SD)	75.6 (5.8)	75.6 (5.3)	75.4 (6.0)	75.8 (6.0)	0.826	75.5 (6.0)	75.3 (5.8)	77.1 (6.5)	0.078
Education, years, mean (SD)	11.6 (2.8)	12.8 (3.1)	11.6 (2.5)	12.3 (3.1)	0.080	11.1 (2.2)	10.5 (1.9)	9.9 (2.4)	<0.001
Living alone, %	23.1	11.9	15.3	26.3	0.001	28.2	27.8	45.1	0.014
Alcohol, %									
daily	24.3	44.4	38.9	33.7		8.7	5.1	5.6	
sometimes	14.6	17.5	20.8	15.8	0.033	12.3	12.7	7.0	0.108
none/rarely	61.1	38.1	40.3	50.5		79.0	82.3	87.3	
Smoking, %									
current	10.3	15.4	15.3	14.7		4.2	7.6	8.5	
former	33.4	54.9	55.6	61.1	0.551	10.0	6.3	19.7	0.014
never	56.3	29.7	29.2	24.2		85.8	86.1	71.8	
Self-perceived chewing ability, %									
can chew anything/most things do not chew much	97.4 2.6	99.0 1.0	97.2 2.8	94.7 5.3	0.015	98.7 1.3	93.7 6.3	93.0 7.0	0.003
Medical history, %									
Hypertension	48.2	53.5	37.5	51.6	0.391	43.0	45.6	59.2	0.021
Hyperlipidemia	33.2	31.1	23.6	20.0	0.026	39.2	36.7	39.4	0.928
Diabetes	13.5	16.4	23.6	14.7	0.986	9.4	13.9	7.0	0.889
Cancer	12.9	15.7	13.9	13.7	0.591	11.0	10.1	11.3	0.988
Stroke	6.3	9.1	5.6	10.5	0.860	3.6	5.1	2.8	0.951
Heart disease	15.1	20.6	8.3	24.2	0.872	9.4	10.1	18.3	0.047
Arthritis	14.4	6.6	9.7	14.7	0.016	22.3	19.0	9.9	0.020
COPD	3.9	2.8	1.4	8.4	0.030	4.2	1.3	7.0	0.562
BMI, kg/m^2^, mean (SD)	23.1 (3.2)	23.5 (2.8)	23.0 (3.2)	23.7 (3.1)	0.833	22.5 (3.3)	22.8 (4.3)	23.2 (3.2)	0.118

SD, standard deviation; BMI, body mass index.

^a^Participants were asked the following question: “How many days per week do you eat at least 2 meals a day with a staple food, main dish, and side dish?”. The frequency was self-reported according to the following four categories: “≤1 d/wk”, “2 or 3 d/wk”, “4 or 5 d/wk”, and “daily”.

^b^*P* values are based on weighted one-way analysis of variance, for continuous variables, or the Mantel-Haenszel chi-square test, for categorical variables.

Table 2 shows the associations of frequency of balanced-meal consumption with frailty (prefrailty and frailty combined). In model 1 (age, sex, and study site-adjusted), frequency of balanced-meal consumption was significantly associated with frailty prevalence. Although adjustment for other covariates somewhat attenuated the association (model 2), it remained statistically significant. As compared with participants with a frequency of daily, those with a frequency of ≤2 or 3 days/week had a higher risk of frailty (odds ratio [OR], 1.79; 95% confidence interval [CI], 1.21–2.64; *P* for trend = 0.007). The results for men and women are also shown in Table 2. In men, those with a frequency of ≤2 or 3 days/week had a higher risk of frailty (OR, 1.74; 95% CI, 1.07–2.84); however, this association disappeared after multivariable adjustment in model 2. In women, after adjusting for covariates, as compared with participants with a frequency of daily, those with a frequency of ≤2 or 3 days/week had a significantly higher risk of frailty (OR, 2.28; 95% CI, 1.24–4.19; *P* for trend = 0.007).

**Table 2.** Odds ratios and 95% confidence intervals for associations of frequency of twice-daily balanced-meal consumption with frailty (prefrailty and frailty combined)

	Frequency of twice-daily balanced-meal consumption			*P* for trend

	Daily	4 or 5 d/wk	≤2 or 3 d/wk	
**Frailty (prefrailty and frailty combined)**				
**All (*n* = 912)**				
Number of cases, %	33.3	38.4	52.4	
Model 1	1.00 (Reference)	1.10 (0.75–1.63)	1.96 (1.36–2.83)	0.001
Model 2	1.00 (Reference)	1.03 (0.69–1.54)	1.79 (1.21–2.64)	0.007
**Men (*n* = 453)**				
Number of cases, %	33.6	33.3	48.4	
Model 1	1.00 (Reference)	0.80 (0.45–1.42)	1.74 (1.07–2.84)	0.055
Model 2	1.00 (Reference)	0.76 (0.42–1.39)	1.55 (0.92–2.62)	0.184
**Women (*n* = 459)**				
Number of cases, %	33.0	43.0	57.7	
Model 1	1.00 (Reference)	1.48 (0.87–2.51)	2.30 (1.32–4.01)	0.002
Model 2	1.00 (Reference)	1.38 (0.79–2.41)	2.28 (1.24–4.19)	0.007

Model 1 was adjusted for sex, age, and study site. Model 2 was adjusted for variables in Model 1 plus education, living alone, smoking and drinking habits, body mass index, self-perceived chewing ability, and medical history (hypertension, hyperlipidemia, diabetes, cancer, stroke, heart disease, arthritis, and chronic obstructive pulmonary disease).

Table 3 shows the associations of frequency of balanced-meal consumption with frailty components. A lower frequency of balanced-meal consumption was associated with higher prevalences of weight loss (OR, 4.10; 95% CI, 1.90–8.85; *P* for trend < 0.001), exhaustion (OR, 6.35; 95% CI, 2.49–16.17; *P* for trend < 0.001), and low physical activity (OR, 1.92; 95% CI, 1.22–3.01; *P* for trend = 0.013). In contrast, no associations were observed for other frailty components, such as muscle weakness and slowness. Analyses stratified by area showed no clear difference in relation to area (data not shown).

**Table 3.** Odds ratios and 95% confidence intervals for associations of frequency of twice-daily balanced-meal consumption with frailty components

	Frequency of twice-daily balanced-meal consumption			*P* for trend

	Daily	4 or 5 d/wk	≤2 or 3 d/wk	
**All (*n* = 912)**				
**Weight loss**				
Number of cases, %	2.5	2.6	10.8	
Model 1	1.00 (Reference)	1.10 (0.35–3.41)	4.42 (2.14–9.12)	<0.001
Model 2	1.00 (Reference)	1.07 (0.33–3.46)	4.10 (1.90–8.85)	<0.001
**Exhausion**				
Number of cases, %	1.7	6.0	9.6	
Model 1	1.00 (Reference)	3.24 (1.27–8.28)	5.21 (2.29–11.86)	<0.001
Model 2	1.00 (Reference)	2.89 (1.07–7.80)	6.35 (2.49–16.17)	<0.001
**Muscle weakness**				
Number of cases, %	16.5	21.9	21.1	
Model 1	1.00 (Reference)	1.40 (0.85–2.29)	1.17 (0.72–1.90)	0.371
Model 2	1.00 (Reference)	1.32 (0.79–2.22)	1.08 (0.64–1.81)	0.600
**Slowness**				
Number of cases, %	7.6	9.3	12.0	
Model 1	1.00 (Reference)	1.15 (0.59–2.23)	1.40 (0.77–2.53)	0.266
Model 2	1.00 (Reference)	1.06 (0.53–2.09)	1.35 (0.72–2.50)	0.370
**Low physical activity**				
Number of cases, %	14.3	16.6	28.3	
Model 1	1.00 (Reference)	0.98 (0.60–1.61)	2.09 (1.38–3.18)	0.002
Model 2	1.00 (Reference)	0.87 (0.52–1.46)	1.92 (1.22–3.01)	0.013

Model 1 was adjusted for sex, age, and study site. Model 2 was adjusted for variables in Model 1 plus education, living alone, smoking and drinking habits, body mass index, self-perceived chewing ability, and medical history (hypertension, hyperlipidemia, diabetes, cancer, stroke, heart disease disease, arthritis, and chronic obstructive pulmonary disease).

To ensure that missing data did not introduce bias to results regarding the association between frequency of balanced-meal consumption and frailty, sensitivity analyses were conducted using five imputed datasets generated with a multiple imputation approach. All results from multiple imputed analyses were similar to those from complete-case analyses (data not shown).

## DISCUSSION

This cross-sectional study of community-dwelling older Japanese showed that a lower frequency of eating balanced meals more than twice a day was associated with increased risks of prefrailty and frailty, after adjustment for potential confounders. To our knowledge, this is the first study of the association between balanced-meal consumption and frailty in community-dwelling older Japanese.

Several studies have assessed the relation between dietary pattern and frailty risk.^14^^–^^16^^,^^32^^–^^36^ Most addressed the effects of the Mediterranean dietary pattern on frailty risk and showed that higher intakes of vegetables, fruits, and fish were linked to lower frailty risk.^14^^–^^16^ However, the Mediterranean dietary pattern is uncommon in Asia and might not be realistic for Asian populations. To our knowledge, only two studies examined the association between dietary pattern and frailty in Asian populations.^35^^,^^36^ A Chinese prospective study found that better diet quality, as assessed using the Diet Quality Index-International, was associated with lower risk of frailty development.^35^ In a Taiwanese cross-sectional study, a dietary pattern derived from the empirical reduced rank regression method was significantly inversely associated with frailty.^36^ This dietary pattern comprised high intakes of fruits, nuts and seeds, tea, vegetables, whole grains, shellfish, milk, and fish. Although direct comparison of past and present results is complicated by the use of different methods to measure dietary pattern, the existing evidence suggests that a diet with adequate energy and optimal protein intake, along with substantial intakes of plant-based and antioxidant-containing foods, such as vegetables, is important in preventing or delaying frailty onset in older adults.

The mechanism linking frequency of balanced-meal consumption and frailty is unclear, but several processes have been suggested. The nutrients most consistently linked to frailty components in observational studies include protein, vitamin D, antioxidant nutrients (including carotenoids, selenium, and vitamins E and C), and long-chain PUFAs.^37^^,^^38^ A main dish that includes fish or meat is the main source of protein. A staple food that includes rice or bread is the main source of carbohydrate. A side dish that includes vegetables, mushrooms, potatoes, or algae is important in supplementing the meal with vitamins, minerals, and dietary fiber. To our knowledge, only two studies have examined diets including balanced meals. One found that more frequent consumption of balanced meals was associated with favorable nutrient intake among Japanese young adults.^21^ Another reported that more frequent consumption of balanced meals was associated with prevention of nutritional deficiencies, such as calcium and vitamin C deficiencies, in Japanese adults aged 40–59 years.^22^ Our preliminary analysis of a subsample (only the participants in the Hatoyama Cohort Study) showed that participants with less frequent balanced-meal consumption consumed lower amounts of nutrients than did those with more frequent balanced-meal consumption; the respective mean intakes (nutrient intakes assessed using self-administrated diet history questionnaire; values energy-adjusted using density methods) for the ≤2 or 3 days/week and daily categories of the frequency of this meal were 15.4% and 16.8% for protein (% energy), 61.1 mg and 71.3 mg for vitamin C, 8.8 µg and 10.4 µg for vitamin D, and 1.5 g and 1.7 g for n-3 PUFA among men, and 16.2% and 17.8% for protein, 66.5 mg and 92.4 mg for vitamin C, 7.8 µg and 11.1 µg for vitamin D, and 1.6 g and 1.8 g for n-3 PUFA among women. These findings support the association between balanced meals and a high-quality diet with satisfactory intakes of important nutrients, which could have a beneficial effect on frailty in men and women.

In the present study, eating balanced meals more than twice daily was significantly associated with lower odds of prefrailty and frailty in women. However, the association between balanced meals and frailty was weak in men and disappeared after multivariable adjustment. The attenuation of the association between balanced meals and frailty after adjustment for medical and lifestyle factors suggests that these covariates largely mediate the association between balanced meals and frailty. To take one example, the proportion of smokers was much higher in men, and evidence suggests that smoking is a predictor of worsening frailty status among community-dwelling adults.^39^ The strong effect of residual confounding attributable to smoking may have obscured the association in men. Future studies will need to explore these sex differences in greater detail.

In the present study, low frequency of balanced-meal consumption was associated with higher prevalences of weight loss, exhaustion, and low physical activity. In particular, we found a strong inverse association of balanced-meal consumption with weight loss and exhaustion. Although the mechanisms underlying this association are unclear, a previous study reported that a protein intake of >1.0 g/(kg/d) protected against weight loss at 1 year in healthy older adults.^40^ Moreover, a previous prospective study reported that vegetable consumption was associated with decreased risks of frailty, exhaustion, and unintentional weight loss.^12^ As described above, balanced meals are associated with higher protein intake and high intake of antioxidants in vegetables, which could prevent unintentional weight loss and exhaustion.

In contrast, no association was observed with other frailty components, such as muscle strength or walking speed. This discrepancy may be partly attributable to the fact that we did not analyze intakes of dairy products and fruits. A previous prospective study reported that higher intake of low-fat dairy products was associated with lower risk of slow walking speed.^13^ In the Fourth Korea National Health and Nutrition Examination Survey, fruit consumption was inversely associated with sarcopenia prevalence.^41^ Because muscle strength and walking speed are central to the definition of frailty developed by Fried and colleagues, further analysis is necessary in order to determine whether these foods are key components of muscle strength and walking speed in older adults.

In the present study population, 63.8% of men and 65.5% of women aged ≥70 years reported daily consumption of two or more balanced meals a day. These prevalences were slightly higher than those reported in a study of national representative data. In the 2015 National Health and Nutrition Survey, Japan, 59.1% of older men and 61.7% of older women (aged ≥70 years) reported daily consumption of at least two balanced meals.^30^ Although the purpose of the nutritional and dietary habits included in the Healthy Japan 21 guidelines is to increase the percentage of individuals who eat balanced meals at least twice a day to 80% by 2022, this target has not yet been reached. In contrast, a previous study reported that the percentage of people who ate a balanced meal at least twice a day was lower in a younger age group (eg, 37.9–45.2% of men and 38.4–51.8% of women aged 20–59 years) than in older age groups.^30^ This result suggests that public health strategies for frailty prevention should focus on younger age groups. Thus, increasing the percentage of adults who eat balanced meals remains a major challenge in public health, and effective interventions are needed.

Major strengths of this study include our use of a sample of community-dwelling elders and adjustment for potentially important confounders. In addition, few studies have examined the role of nutrition on frailty in Asian populations. In Japan, a previous multicenter cross-sectional study enrolled only women^4^^,^^17^^,^^18^; however, the current study included both sexes, and the associations were similar for men and women. Finally, the recommendation to eat balanced meals is easy to put into practice in daily life.

The limitations of the present study also warrant mention. First, an association derived from a cross-sectional study design does not necessarily indicate causality: we cannot exclude the possibility that diet changed because of exhaustion or low physical activity. However, frailty is hypothesized to be preceded by a cycle of frailty,^25^ in which an unbalanced diet may trigger a cascade leading to frailty. The prospective studies mentioned above appear to show this direction in the association.^12^^,^^16^^,^^32^ Second, questionnaire data on the frequency of balanced-meal consumption may not be fully valid. To assess reproducibility, we calculated the Spearman correlation coefficients for the frequency of eating balanced meals at least twice daily from questionnaires separated by a 1-year interval (among participants in the Hatoyama Cohort Study only). The reproducibility was fair (Spearman correlation coefficient, 0.33). Third, because of limitations in the data, we used a modified version of the validated Fried phenotype. Levels of physical activity referred only to frequency of exercise, without information on exercise intensity or duration. This might have led to overestimation of frailty prevalence. Finally, despite adjustment for numerous covariates, we cannot exclude the possibility of residual confounding by uncontrolled factors, such as cognitive status and depression.

### Conclusions

In conclusion, a lower frequency of eating balanced meals at least twice a day was associated with increased prevalences of prefrailty and frailty, after adjustment for potentially important confounding factors. The present study provides additional evidence that dietary assessment of older people helps them maintain optimal health and fosters successful aging. The observed associations require confirmation in prospective studies.
